# Reactivation of telomerase in cancer

**DOI:** 10.1007/s00018-016-2146-9

**Published:** 2016-02-04

**Authors:** Semih Can Akincilar, Bilal Unal, Vinay Tergaonkar

**Affiliations:** 1 Institute of Molecular and Cell Biology (IMCB), A*STAR (Agency for Science, Technology and Research), Proteos, 61, Biopolis Drive, Singapore, 138673 Singapore; 2 Department of Biochemistry, Yong Loo Lin School of Medicine, National University of Singapore (NUS), Singapore, 117597 Singapore; 3 Centre for Cancer Biology, University of South Australia and SA Pathology, Adelaide, Australia

**Keywords:** TERT reactivation, NF-κB, *Tert* promoter mutation, Transcription, Cancer

## Abstract

Activation of telomerase is a critical step in the development of about 85 % of human cancers. Levels of *Tert*, which encodes the reverse transcriptase subunit of telomerase, are limiting in normal somatic cells. *Tert* is subjected to transcriptional, post-transcriptional and epigenetic regulation, but the precise mechanism of how telomerase is re-activated in cancer cells is poorly understood. Reactivation of the *Tert* promoter involves multiple changes which evolve during cancer progression including mutations and chromosomal re-arrangements. Newly described non-coding mutations in the *Tert* promoter region of many cancer cells (19 %) in two key positions, C250T and C228T, have added another layer of complexity to telomerase reactivation. These mutations create novel consensus sequences for transcription factors which can enhance *Tert* expression. In this review, we will discuss gene structure and function of *Tert* and provide insights into the mechanisms of *Tert* reactivation in cancers, highlighting the contribution of recently identified *Tert* promoter mutations.

## Telomere and telomerase holoenzyme

Telomeres are conserved, repetitive sequences located at the ends of eukaryotic chromosomes which protect the integrity of genomic DNA [1, 2]. DNA polymerase is unable to replicate the 5′ ends of chromosomes, hence, cells require a RNA dependent DNA polymerase called telomerase to synthesize DNA on the lagging strand [3–5]. Telomerase activity is tightly regulated and seen mainly in germ cells, stem cells and some immune cell types which have high proliferative needs. In contrast, somatic cells do not display detectable telomerase activity [6]. As a result, the chromosomes of normal somatic cells shorten 50–200 bp each replication at the telomeres due to the problem of end replication. Thus, somatic cells are eventually burdened with DNA damage, replication crisis, cellular senescence or apoptosis and can divide only limited number of times [7], whereas cells that have active telomerase possess unlimited proliferative potential.

Telomerase was discovered by Carol Greider and Elizabeth Blackburn in 1984 from ciliate Tetrahaymana [5]. Telomerase holoenzyme is comprised of a catalytic subunit, hTERT (human telomerase reverse transcriptase) that has reverse transcriptase activity and an RNA component, hTR (human telomerase RNA component) which primes DNA synthesis from telomere repeats. The three dimensional structure of human telomerase is yet to be fully understood. To date, it remains a challenge to purify and crystallize the entire telomerase complex due to its low abundance (estimated to be approximately 20–50 molecules of telomerase per HEK-293 cells [8], and estimated ~250 molecules even in the highly telomerase active cancer cell lines [9]), insolubility problems, and also the requirement of substantial enrichment. Biochemical characterization, however, suggests that hTERT and hTR are sufficient to recapitulate telomerase activity in vitro; however, other cellular factors may be required for basal in vivo activity. Mass spectrometric studies and reconstitution approaches suggest that telomerase has a minimum molecular weight of 650–670 kD and each component appears as a dimer in the core complex including hTERT 127kd, hTR 156kd, DKC1 (dyskerin) 57 kD and Nop10 9 kD [10]. Cohen et al. also demonstrated that telomerase can occur as a dimer [8], but in a recent study Wu et al. showed that telomerase can also function as a monomer and is able to add telomeric ends [11]. Furthermore, proteins like Pontin, Reptin, Gar1, Nhp2, and Tcab1 were shown to be transiently associated with the telomerase core complex and are thought to be required for proper telomerase assembly and recruitment to chromosomes [12, 13]. Current knowledge proposes that the limiting factor for telomerase activity is the level of TERT which is kept under tight transcriptional control. Expression of *Tert* mRNA in somatic cells is sufficient to reconstitute telomerase activity [14–16] and expression levels of *Tert* always show strong correlation with telomerase activity [17, 18]. Although the level of RNA component of the telomerase holoenzyme hTR is high in all tissues regardless of telomerase activity [19] it is generally expressed at a lower level in normal somatic cells as compared to cancer cells [20]. Furthermore, half-life experiments for hTR showed a higher turnover rate (~5 days) in somatic cells, whereas the half-life is proposed to be between 3 and 4 weeks in cancer and highly proliferative stem cells [20]. Most cancer cells have more than 10,000 hTR molecules per cell whereas quantification studies by competitive RT-PCR assays show that hTert mRNA range between 1 and 30 molecules in human cancer cells [21]. Other quantification studies determined that TERT protein numbers range between 600 and 700 molecules per cell in HeLa cells [22]. It was also reported that the half-life of hTert mRNA is only 2–3 h [23], while the half-life of active telomerase complex appears to be approximately 24 h according to activity assays [24]. These observations indicate that hTR is essential for the activity of telomerase; however, the limiting factor for telomerase activity is strictly dependent on transcription of hTert mRNA with confounding effects on the stability of the complex by yet unidentified factors. On the other hand, it is noteworthy that hTR can be the limiting factor for telomerase activity in some cases like in the fibrosarcoma-derived HT1080 cells [25]. In general, these results underlie the complexity of telomere maintenance but overall any defect in telomere maintenance failure results in general genomic instability. In particular, DNA damage, increased cellular senescence and organ degeneration are key features of aging phenomena due to telomere shortening [26–28].

## Telomerase reactivation in cancer cells

*Tert* expression is reactivated in ~85 % of all cancers [29]. Recently numerous reports have indicated oncogenic effects of TERT independent of its role in telomere elongation [30–32]. Telomere length in mice (20–50 kb) is greater than in humans (5–10 kb) [33–35]. However, *Tert* expression is upregulated in murine breast [36] and skin [37] tumors despite their long telomeres. These results suggested that TERT could be playing other roles in cancer. Moreover, overexpression of *Tert* resulted in increased cell proliferation in mammary carcinomas [38] and epidermal tumors [39] in mice. Similarly, increased expression of *Tert* initiated T cell lymphomas in mouse thymocytes [40] without significant changes in telomere length, thereby supporting its telomere independent role [32]. Moreover, in human cancer cell lines, knocking down *Tert* resulted in rapid decreases in cell proliferation and growth [41]. Mechanistically, TERT was shown to indirectly associate with promoters of NFκB target genes interleukin (IL)-6, tumor necrosis factor (TNFα) and IL-8, which are critical for inflammation and cancer progression, to increase expression [42]. Expression of catalytically inactive *Tert* in mouse model led to the activation of hair follicle stem cells and induction of hair growth [43]. Although the major function of telomerase is thought to be telomere elongation, accumulating evidence has suggested that it can modulate expression of various genes including target of Wnt/β-catenin [44] and NFκB signaling [42] which affect cancer progression and tumorigenesis [45]. High throughput genomic and transcriptomic analysis has revealed that TERT can regulate expression of about 300 genes involved in cell cycle regulation, cellular signaling, and cell proliferation [46]. Non-canonical roles of telomerase have been discussed previously [31, 32], hence in this review we will be focusing on *Tert* re-activation via regulation of *Tert* transcription.

## Structure of *Tert* gene

The hTert gene is 42-kb long and located on chromosome 5 with 16 exons. The reverse transcriptase domain is coded by exons 5–6–7–8–9. It has been suggested that the telomerase transcript containing 16 exons can be spliced into 22 isoforms [47] but only full length *Tert* transcript possesses reverse transcriptase activity essential for elongating telomeres [48, 49]. The most commonly studied isoforms in cancer cell lines involve exon 6–9 that partially encode the reverse transcriptase domain [49]. Alternative spliced isoforms within this region produce isoforms named minus alpha, minus beta and minus alpha/beta. Minus alpha isoform is spliced from a 36-bp acceptor site into exon 6 that creates dominant-negative protein without reverse transcriptase activity [49]. Overexpression of the minus alpha form resulted in inhibition of telomerase which leads to either cell death or senescence [50]. The minus beta isoform has a stop codon in exon 10 due to a frameshift after skipping exons 7 and 8. This isoform is the most common form among cancer cell lines. Although minus beta *Tert* is subjected to non-sense mediated mRNA decay due to a premature stop codon, it has been shown that its transcripts can be translated into protein [51]. Listerman et al. have reported that overexpression of the minus beta form competed for hTR with endogenous telomerase activity. In their study, Listerman and colleagues reported that overexpressed minus beta form conferred a growth advantage in breast cancer cells by protecting cells from cisplatin-induced apoptosis [52]. However, it remains to be determined why cancer cells preferentially express the catalytically inactive minus beta spliced variant of *Tert*. *Tert* isoforms can be expressed in a given cell simultaneously; however, ratios may differ among different cell types according to external stimuli [51]. Deletions/insertions distant from the RT domain also result in truncated forms of TERT protein, the functions of which are not fully understood and need careful characterization.

**Fig. 1:**
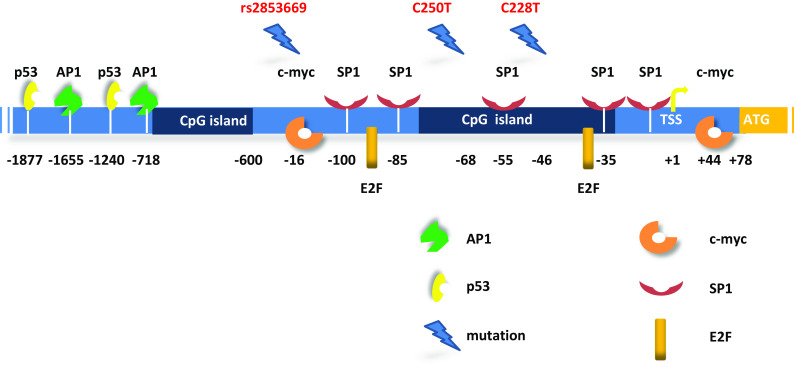
Schematic of *Tert* promoter region with regulatory protein binding sites and de-novo mutations. The +1 and +78 (ATG) indicates transcription start site (TSS) and first codon for TERT protein, respectively. *Dark blue* regions correspond to CpG islands. Highly recurrent mutations C228T and C250T are shown at −46 and −68 positions from TSS. The rs2853669 polymorphism is shown in ETS2 binding site (−167 from TSS). The sites on the promoter are not precisely scaled

The *Tert* promoter has complex regulation dynamics whereby multiple transcriptional regulatory elements play functional roles in different contexts either individually or interactively. Although the *Tert* promoter does not have typical transcription regulatory elements like TATA and CAAT boxes [53], it contains recognition sequences for multiple important transcription factors such as p53, p21, SP1, ETS, E2F, AP1, HIF1 and c-myc (Fig. 1) [54–62]. Increasing evidence shows that the level of telomerase activity is primarily regulated through levels of *Tert* which are primarily controlled by transcription of *Tert* gene. Although the transcription factors stated above may alter the ability to regulate *Tert* transcription under specific cell type and physiological conditions, none of them are sufficient on their own to promote immortalization of somatic cells [63].

The *Tert* promoter region contains E-boxes and GC-boxes which are the consensus binding sites for transcription factors c-myc and SP1, respectively. These factors are known to regulate many cellular events like cell growth, apoptosis, and chromatin remodeling. Sp1 and c-myc cooperatively regulate *Tert* expression in a cell type specific manner [58]. One of the prominent characteristics of *Tert* promoter is its high GC-rich content without a typical TATA box or a CCAAT box. There are five GC-boxes in the proximal *Tert* promoter which serve as SP1 binding port and two E-boxes (CACGTG) located in the −165 and +44 regions which are targetable by c-myc and\or max proteins through their helix–loop–helix leucine zipper domains [58]. Luciferase reporter assays reveal that deletion of the E-box at the −165 position results in 60 % reduction in transcriptional activity in C33A and ME180 cells but not in SiHa cells. Interestingly, mutating the other E-box domain at position +44 led to a 60 % reduction only in ME180 cells. Expression of estrogen, a sex hormone, exhibits a strong concordance with *Tert* transcription. Mutations in the estrogen receptor (ER) binding sites of *Tert* promoter dramatically reduced *Tert* transcription. Moreover, c-myc is a known target of ER and some other growth factors. Kyo et al. demonstrated that the effect of estrogen was completely abrogated upon mutating E-boxes in proximal *Tert* promoter [57]. Furthermore, mutating the GC-box, located −32 from TSS, reduced transcription by 20–40 % in all cell lines and a 90 % reduction was detected when all SP1 binding sites were mutated [58]. We can thus conclude that Sp1 binding sites (GC-boxes) are essential for *Tert* transcriptional activity and E-boxes can accelerate transcription in a cell-type specific manner. E2F transcription factors are involved in cell cycle regulation and DNA synthesis [64, 65]. Ectopic expression of E2F1 repressed *Tert* promoter activity through inhibition of SP1 binding to DNA in cancer cells. In contrast, overexpression of E2F1 in normal human fibroblasts increased *Tert* promoter activity through a non-canonical E2F1 site which is located –51 to –88 [66]. All these data suggest that E2F1 has a dual role for regulating *Tert* expression that requires further investigation.

The tumor suppressor gene p53 has two binding sites −1240 and −1877 upstream of the transcription start site of the *Tert* promoter (Fig. 1). Overexpression of p53 together with SP1 leads to suppression of *Tert* expression while siRNA silencing of p53 delays senescence but is insufficient to drive cells to an immortal state [67, 68]. Overexpression of *Tert* is not sufficient to drive cells to the immortal state as well. *Tert* expression and telomerase activity could be enhanced by suppression of p53; however, both actions are required for immortalization of primary human ovarian surface epithelial cells [67, 68].

NFκB is a key regulator of innate and adaptive immunity which is essential for host defense [69, 70]. Activated NFκB activates the expression of target genes that are responsible for cell survival, proliferation, differentiation of cells and for mounting appropriate immune responses for host defense [71]. NFκB was also reported to be associated with tumorigenesis and chemoresistance [71, 72]. The *Tert* promoter contains a NFκB binding site upstream of its translation start site. A significant increase in *Tert* expression was observed through NFκB activation [73]. Moreover, NFκB can mediate TERT translocation to the nucleus from the cytoplasm whilst IKK2 inhibitors can reverse this effect in MM.1S cells [74].

The PI3K-AKT pathway regulates various cellular events such as proliferation and differentiation which are important in tumorigenesis [75]. It has been reported that *Tert* expression was increased by receptor tyrosine kinases and VEGF which are controlled by PI3K in ovarian cancer cells [76]. Moreover, *Tert* expression is increased depending on two VEGF isoforms, VEGF165 and VEGF189, which are also positively regulated upon *Tert* expression regardless of its telomeric function in HeLa cells [77]. Under physiological conditions, all of the molecules listed above are responsible for specific cellular processes and almost all somatic cells can achieve these processes without reactivating their *Tert* gene. However, multiple changes and impairments evolve during cancer progression that cause chromosomal re-arrangements, point mutations, deletions/insertions, translocations and copy number changes.

The *Tert* gene has a GC rich promoter which contains three CpG islands [53, 78]. Methylation of gene promoters is generally known to repress transcription; however, several studies revealed complex methylation patterns for the active/inactive *Tert* promoter. The *Tert* promoter region has a CpG island (positions −1100 to +150), that is mostly hypermethylated through specific DNA methyltransferases (DNMTs) in cancer cells [79]. The *Tert* promoter between −150 and +150 represents a usual pattern of gene expression. Absence of methylation causes constitutive expression particularly in this region, hypermethylation of 5′ *Tert* promoter prevents binding of methylation sensitive CTCF repressor to the first exon; however, partial hypomethylation of core promoter is required for *Tert* transcription. Thus, *Tert* promoter methylation represents a unique model for transcription in which hypermethylation of cytosine islands causes inhibition of *Tert* expression and this differs among different cell types [79, 80]. Hypermethylation of the *Tert* core promoter results in low telomerase activity and better survival rate in B cell chronic lymphocytic leukemia [81]. On the contrary, several studies reported that hypermethylation in telomerase active cancer cells and hypomethylation of normal tissues may inhibit binding of repressor elements to the *Tert* promoter region and enhance transcriptional activity [78, 80, 82–84]. A comprehensive study by Renaud et al. demonstrated that the *Tert* promoter in most cancer cell lines is heavily methylated between −500 and −600 bp upstream of TSS; however, they tend to be partially methylated at TSS region [79]. The Myc/Max proteins are responsible for recruiting Histone Acetyl transferases (HATs) to the promoter regions where they can bind through their consensus sequences [85]. Increased acetylation of H3 and H4 histones creates more open chromatin structure, thereby enhancing the accessibility of other transcription regulatory elements. However, Mad proteins could bind to the same E-boxes and heterodimerize with Max proteins that act antagonistically to inhibit transcription [86].

In conclusion, *Tert* promoter specific methylation analysis revealed a complex/unusual methylation pattern for the promoter region, indicating that distal and proximal promoter regions have different methylation patterns that can be partially modified due to activation or inactivation of the *Tert* gene. These results highlight importance of chromatin modifiers, especially the roles of histone methyl transferases and demethylases in transcription dynamics.

## *Tert* promoter mutations in cancer

**Fig. 2:**
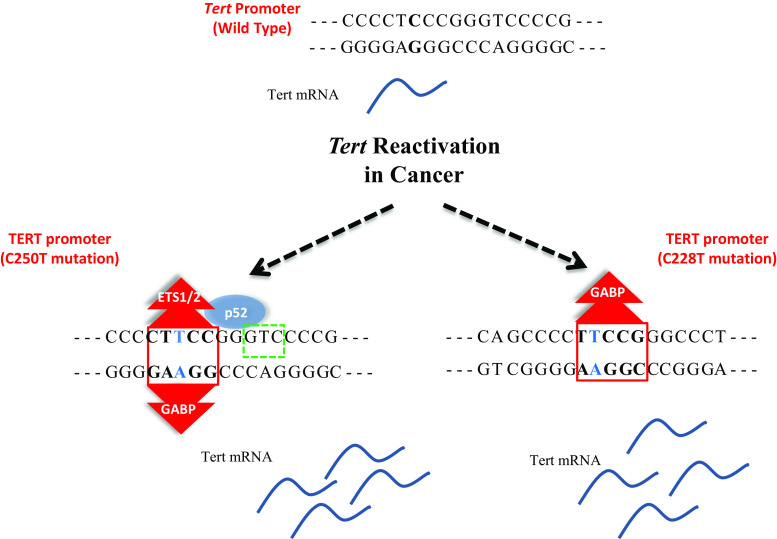
Highly recurrent mutations C228T and C250T create novel ETS binding sites in *Tert* promoter. *Tert* promoter with wild type, C250T and C228T are shown with their reported binding partners. *Red* and *green rectangular boxes* indicate ETS and p52 consensus sites on *Tert* promoter, respectively. ETS1/2 and p52 binds *Tert* promoter harboring C250T mutation while GABP binds to *Tert* promoter bearing either C250T or C228T mutation, resulting in increased *Tert* mRNA expression

Point mutations in the *Tert* promoter are highly recurrent in cancer cases including glioblastoma, melanoma, urothelial, bladder, hepatocellular carcinoma, and thyroid cancers [87–91]. These mutations generally create new consensus motifs for transcriptional regulators like ETS/TCF factors and are associated with increased *Tert* mRNA (Fig. 2). Therefore, point mutations in the *Tert* promoter could unravel a novel mechanism for *Tert* reactivation in cancer cells. Recurrent mutations have been identified in Chr.5:1,295,228(C>T) or (CC>TT), Chr.5:1,295,250 C>T, Chr.5:1,295,242_1,295,243CC>TT mutations in 19 % of cancers [90].

Alternative lengthening of telomeres (ALT) is a recombination-based mechanism that is activated in the absence of telomerase activity. ALT recombination is stimulated by the signals of double-strand DNA break and meiotic HOP2-MND1 heterodimer induces RAD51 and DMC1 mediated recombination [92]. ALT is observed frequently in sarcomas (25–60 %) [93], brain tumors (10–25 %) [93], and pancreatic neuroendocrine tumors (40 %) [92], but is rare in colon, breast, lung, prostate and pancreas cancers. Generally, cancers with ALT show poor prognosis as compared to telomerase activity. However, glioblastomas with ALT represent two- to threefold longer survival [92, 94]. Recently it was also shown that mutations in ATRX or DAXX genes cause increase in non-coding RNA TERRA expression which is known to inhibit telomerase activity [95–98]. Furthermore, TERRA can induce ALT through R-loop formation in telomeres [92]. ATRX and DAXX alter telomeric chromatin dynamics that function as a chromatin remodeler and histone chaperone, respectively. Therefore, cells having these mutations depend on ALT for telomere maintenance. Interestingly, ATRX/DAXX genes and *Tert* promoter mutations are mutually exclusive [87]. Besides, there are subpopulations that display both ALT and telomerase mechanisms for telomere maintenance [99] which could be a temporary/transient stage before final commitment to ALT and/or telomerase as the major telomerase mechanism depending on the cellular context and other signaling milieu. However, there is very little knowledge regarding the mechanism by which cells can utilize both mechanisms or how they choose between ALT or telomerase as a preferred mechanism to maintain their telomeres.

Hepatocellular carcinomas (HCCs) are the third leading cause of cancer deaths and are mostly associated with hepatitis B or C virus infection while a minority is associated with alcoholic cirrhosis, obesity and rare genetic diseases [100, 101]. Recently, somatic mutations in the *Tert* promoter region have been reported in HCC. Killela et al. reported that 44 % of HCC tumor samples have *Tert* promoter mutations (27/61) and the mutations tend to occur relatively early during tumorigenesis [87]. Other teams have also identified these mutations and, according to recent reports, *Tert* promoter mutations are less frequent in Eastern countries (29 to 31 % of HCC), whereas 54–60 % of HCC are mutated in Western countries [87, 102–104]. The most frequent *Tert* promoter mutations in HCC (93 %) were observed at the first hot spot and consist of G to A (−124G>A) mutations. While mutations in the second hot spot −146 bp G to A substitutions (−146G>A) were significantly less frequent than those found in melanoma (46 %) [105]. Interestingly, *Tert* promoter mutations are more frequent in older patients and significantly associated with activating mutations of the beta catenin pathway [103, 105]. These data suggest that aberrant activation of the β-catenin pathway associated with *Tert* promoter mutations can be one of the mechanisms of telomerase reactivation in HCC. Taken together, these data suggest that somatic mutations in the *Tert* promoter region are one of the most frequently observed genetic alterations in human HCC. Although *Tert* amplification and insertion of HBV in the *Tert* promoter has been considered as the alternative mechanism leading to telomerase reactivation in HCC [106, 107], identification of *Tert* promoter mutations in HCCs has opened new insights into telomerase reactivation and telomere maintenance in liver carcinogenesis.

Bladder cancers represent the most common urinary tract cancer worldwide [108, 109] and recent studies have showed that *Tert* promoter mutations are the most common mutations in all stages and grades of bladder cancer with an even distribution [110–112]. In addition, the frequency of *Tert* promoter mutations detected in these studies was higher than any earlier reported genetic alteration in any gene in bladder cancer [112, 113]. *Tert* promoter mutations were detected in both low-grade and high-grade tumors of bladder cancer [90] and, in particular, the −124C>T mutation was detected as the most frequent alteration in 175 (53.5 %) tumor samples [112]. In contrast, the prevalence of ALT in bladder cancer is as low as 1 % in 188 samples [114]. Intriguingly, *Tert* promoter mutations in conjunction with the identified common polymorphism have effects on both survival and recurrence in bladder cancer. The common polymorphism rs2853669 from −245 bp ATG start site in the *Tert* promoter acted as a modifier of survival and recurrence in bladder cancer patients. Bladder cancer patients who do not harbor a variant allele of rs2853669 showed almost twofold reduction in survival and increased disease recurrence. Mechanistically, the variant allele of polymorphism disrupts a pre-existing non-canonical ETS2 binding site in the proximal region of the *Tert* promoter, adjacent to an E-box (Fig. 1). This mechanism is in contrast to the two highly recurrent *Tert* mutations which generate an ETS/TCF binding site [112]. Disruption of the ETS2 binding site in *Tert* promoter or silencing of ETS2 in breast cancer cells has been shown to result in decreased *Tert* expression and cell proliferation due to the disabling of c-Myc binding to the E-box in *Tert* promoter [62]. Moreover, the variant allele of the SNP has been previously shown to affect telomerase expression and telomere length maintenance in non-small lung cancers [115]. *Tert* promoter mutations in combination with the polymorphism can have the potential to serve as clinical biomarkers for prediction of survival and recurrence in bladder cancer patients [112].

Another cancer type that harbors *Tert* promoter mutations is urothelial cancers (UC). Borah et al. reported that when compared to −146C>T mutation, −124C>T mutation is a frequently altered lesion in 23 UC cell lines derived from different stages and grades, including muscle invasive and non-invasive tumors. These promoter mutations provide increased *Tert* mRNA levels with an 18-fold increase in median value according to qRT-PCR analyses. Although most of these transcripts were found to be alternatively spliced variants of *Tert* which lack functional reverse transcriptase activity, both TERT protein and telomerase enzymatic activity were higher in tumor samples harboring promoter mutations. However, compared to the 18-fold increase in *Tert* mRNA level, they reported much more modest 2-fold increases in the TERT protein and telomerase activity that may indicate higher expression of inactive *Tert* isoforms [91]. These results revealed that *Tert* promoter mutations are an effective strategy to boost the level of telomerase activity in UC which do not employ ALT [114]. It remains to be determined why cancer cells express catalytically inactive TERT isoforms and whether expression of specific isoforms would change in cancers harboring promoter mutations.

Thyroid cancer is an endocrine malignancy that can be classified into two common groups: papillary (~85 %) and follicular (~10 %) thyroid cancers. C228T mutation was observed in 30 of 257 (11.7 %) papillary thyroid cancers (PTC) and 9 of 79 (11.4 %) follicular thyroid cancers (FTC) while interestingly, no mutations were detected in benign thyroid tumors (0 of 85). C250T mutation was uncommon in all groups of thyroid tumors, but is mutually exclusive with C228T mutation. BRAF (v-Raf murine sarcoma viral oncogene homolog B) mutation (V600E), which activates the MAPK pathway, is commonly observed in thyroid cancers [116]. It is also known that cancers harboring BRAF mutation are more aggressive than WT counterparts [117]. *Tert* C228T mutation was correlated with BRAF mutation and it was reported that 19 of 104 cases (18.3 %) coexist in PTC while 11 of 153 (7.2 %) BRAF negative tumors exclusively harbor C228T mutation. In a separate study, BRAF mutation was found to exist in 19 of 30 (63.3 %) C228T mutant tumors, and 85 of 227 (37.4 %) C228T negative tumors [118]. Similarly, Vinagre et al. investigated *Tert* promoter mutations in large thyroid cancers categorized as normal, benign and malignant lesions. *Tert* promoter mutations were only detected in malignant lesions in 11 % of PTCs, 14 % of FTCs, 21 % of poorly differentiated thyroid carcinomas and 13 % of anaplastic carcinomas. The majority of these mutations were C228T. Correlation analysis revealed that *Tert* promoter mutations were highly associated with larger tumor size, older patients, lymph node metastasis and BRAF (V600E) mutation. *Tert* mRNA was also significantly higher in patients with BRAF and *Tert* promoter mutation [90]. These results indicate that *Tert* promoter mutation is prevalent in more aggressive tumors.

Gliomas are the most common cancer of the central nervous system (CNS), with four major subtypes including primary glioblastoma, astrocytoma, oligodendriogliomas and oligoastrocytomas [119]. Primary glioblastomas are the most common glioma among adults with very poor survival rates. It was shown that 83 % of the 78 tumors analyzed harbored *Tert* promoter mutations which were correlated with high *Tert* expression levels and poorer patient survival [87]. Remarkably, EGFR amplification, a common phenomenon in GBM tumors, was observed to be mutually exclusive with *Tert* mutations [87]. Astrocytomas are another frequently occurring glioma subtype. This group displays rare *Tert* promoter mutations (10 %); however, IDH1 and IDH2 mutations (75 %) as well as ATXR mutations (70 %) are common, which is also correlated with the high prevalence of ALT (63 %) in these gliomas. In contrast to astrocytomas, oligodendrogliomas frequently (78 %) harbor *Tert* promoter mutations. Interestingly, more than 90 % of this glioma subtype seems to strictly depend on mutations either in ATRX gene or *Tert* promoter for telomere maintenance, indicating the requirement of these genetic alterations for tumorigenesis of this subtype. Oligoastrocytomas have features of both astrocytomas and oligodendriogliomas and have a 25 % frequency of *Tert* mutations [87].

Recently it was reported that 15 % of grade II and III glioblastoma patients had *Tert* promoter mutations in their genome; however, the mutation rate increases dramatically in grade IV patients to 76 % [90]. Patients with *Tert* promoter mutations in combination with common gene alterations found in gliomas including IDH mutations and 1p19q deletion showed poorer survival rate as compared to patients without *Tert* mutations [120]. This is a clear indication that these promoter mutations increase the malignancy of gliomas through *Tert* activation.

Although a majority of neuroblastoma tumors possess *Tert* promoter mutations, alternative genetic alterations like copy number increase and *Tert* gene re-arrangements leading activation of *Tert* have been observed in cancer patients. Genome wide sequencing analysis of neuroblastoma patients revealed recurrent *Tert* gene re-arrangements occurring at 5p15.33 region leading to a juxtaposition of the *Tert* promoter with strong enhancers. *Tert* re-arrangement was observed in 13 % (28 of 217) of high-risk neuroblastoma tumors. Patients with these re-arrangements had increased *Tert* mRNA expression and poor survival rate. Moreover, multiple active enhancer clusters were detected in the translocated regions adjacent to *Tert* rearrangements indicating that translocation of *cis*-acting regulatory elements can cause up-regulation of *Tert* expression [121]. These results suggest that genomic rearrangements rather than *Tert* copy number changes could be the major cause of aberrant *Tert* expression in neuroblastoma in addition to *Tert* promoter mutations.

Similar observations have been shown in melanoma patients and cell lines. Melanomas frequently harbor mutations in oncogenes like BRAF, NRAS (neuroblastoma RAS viral oncogene homolog), KIT (v-kit Hardy–Zuckerman 4 feline sarcoma viral oncogene homolog) and tumor suppressors CDKN2A and PTEN. These mutations occur according to cancer stage. Recently a high frequency of *Tert* promoter mutations was reported in melanoma patients and cell lines by several groups [90, 122–124]. Horn et al., observed *Tert* promoter mutations in a melanoma-prone family where all members developed melanomas at early ages. Furthermore, the same group reported that 125 of 168 (74 %) metastatic melanoma cell lines carry *Tert* promoter mutations which are located 124 (C>T), (CC>TT), 138 and 146 bp upstream from the ATG start codon with frequencies of 27.4, 4.2, 4.8 and 38.1 %, respectively [122]. Whole genome sequencing analyses revealed that *Tert* promoter mutations are the most frequent mutations after BRAF and NRAS genes in melanomas [123]. Both groups observed two- to fourfold increases in *Tert* expression by Luciferase reporter assays due to novel ETS site generation upon mutations [122, 123]. It is noteworthy that *Tert* mutations are more common in patients with BRAF and/or NRAS mutations [124] and *Tert* mRNA levels are higher when *Tert* and BRAF mutations coexist [90].

Recently two independent groups have studied binding of ETS factors on mutated sites. Bell et al. identified several ETS factors such as ELF1, ETS1 and ETV4 which can bind to the mutation site significantly; however, GABP was enriched more in mutation regions together with PolII (Fig. 2). In addition, chromatin immunoprecipitation experiments for GABP did not show enrichment in K562, A549, HeLa, MCF-7 cells which do not harbor the *Tert* promoter mutation. GABP knockdown also led to a rapid decrease in *Tert* transcription [125]. Li et al. identified binding partner p52 for ETS factor—ETS1/2 to drive *Tert* transcription in cells containing C250T *Tert* mutation (Fig. 2). They showed that non-canonical NFκB signaling is necessary to drive *Tert* transcription particularly in C250T mutant *Tert* promoter by direct interaction with ETS factor. Consistent with their biochemical data, knock down of p52 during non-canonical NFκB activation abolished tumorigenesis in C250T-mutant glioblastoma cells transplanted mouse models. C250T mutation creates a half site NFκB consensus sequence (5′-GGGGG-3′ or 5′-GGAA-3′) and increased p52 binding was observed in the mutant promoter as compared to WT *Tert* sequence in electrophoretic mobility shift assays (EMSA). The group further demonstrated that *Tert* expression increased upon binding of p52 to the novel half site in cells harboring C250T but not C228 mutation. Moreover, ETS1/2 heterodimerized with p52 at C250T region and cooperatively activated *Tert* gene expression, thereby demonstrating the non-canonical role of NFκB in telomerase reactivation in cancer cells harboring *Tert* promoter mutations [126].

## Conclusion

Reactivation of telomerase has been considered as a strategy for telomere maintenance and is a major hallmark of cancer. Telomerase reactivation mostly depends on the amount of TERT in the cell since there are sufficient amounts of other telomerase complex molecules as summarized above. Therefore, increases in TERT could be substituted by increasing gene copy number as in HeLa cells (five copies), overexpression of oncogenes that can bind its promoter for transcription like c-Myc or using alternative splicing to form catalytically active/inactive proteins.

More importantly, *Tert* promoter mutations which create new consensus sequence for ETS and NFκB binding result in increased transcription of *Tert* mRNA. It is also noteworthy that these mutations could lead to chromatin conformation changes by novel short, middle and long distance interactions, which could, in turn, directly regulate the *Tert* gene and possibly other genes simultaneously. Since these mutations are highly recurrent in many cancer types and occur at high frequency, the mechanisms underlying regulation of *Tert* expression at its promoter need to be deciphered. We can speculate that the presence of these mutations could modulate core transcriptional machinery by recruiting additional factors to the *Tert* promoter to regulate expression of specific isoforms of *Tert* transcript preferentially in cancers. Furthermore, it would be interesting to determine why mutations occur persistently in the −146 and −124 positions in a wide range of various cancer types. Recently, Chiba et al. revealed that introducing any of the three most frequent *Tert* promoter mutations using CRISPR/Cas9 genome editing in human embryonic stem cells did not increase *Tert* expression, activity or telomere length. However, when these engineered stem cells were differentiated to either fibroblast or nerve cells, all of the *Tert* promoter mutations, without any additional oncogenic mutations, prevented silencing of the *Tert* promoter and resulted in enhanced *Tert* expression, telomerase activity, telomere length and growth comparable to cancer cell lines [127]. These results indicate that *Tert* promoter mutations do not affect already active promoters but prevent proper silencing of the *Tert* gene in differentiated cells. Recently, it was shown that cancer cell lines harboring *Tert* promoter mutations represent display histone marks [128]. It would be intriguing to speculate that the presence of *Tert* promoter mutations may affect recruitment of epigenetic modulators and enhancers to the *Tert* promoter to drive *Tert* expression.

Since *Tert* promoter mutations are not present in stem and healthy proliferating cells, it will be crucial to decipher the mechanistic pathways which regulate *Tert* gene silencing and understand how they are affected by these promoter mutations. It will also be a wise strategy to identify specific therapeutic approaches targeting only *Tert* promoter mutations so that only tumor cells but not telomerase positive stem cells are eliminated in cancer patients.
